# Patient-reported outcome measures for systemic lupus erythematosus: an expert Delphi consensus to guide implementation in routine care

**DOI:** 10.1186/s41927-024-00401-x

**Published:** 2024-07-16

**Authors:** Isabel Castrejón, Laura Cano, María José Cuadrado, Joaquín Borrás, Maria Galindo, Tarek C. Salman-Monte, Carlos Amorós, Carmen San Román, Isabel Cabezas, Marta Comellas, Alejandro Muñoz

**Affiliations:** 1 Departamento de Medicina, Servicio de Reumatología, Hospital General Universitario Gregorio MarañónInstituto de Investigación Sanitaria Gregorio MarañónUniversidad Complutense de Madrid, C. del Dr. Esquerdo, 46, 28007 Madrid, Spain; 2 Enfermería Reumatología, H. Regional de Málaga, Málaga, Spain; 3 Servicio de Reumatología, H. Clínica Universitaria de Navarra, Madrid, Spain; 4 Farmacia Hospitalaria. H. de Sagunto, Valencia, Spain; 5 Servicio de Reumatología, H. 12 de Octubre, Madrid, Spain; 6 Servicio de Reumatología, H. del Mar, Barcelona, Spain; 7 GlaxoSmithKline, Madrid, Spain; 8 Outcomes’10, Castellón de La Plana, Spain; 9 Servicio de Reumatología, H. Universitario Virgen del Rocío, Sevilla, Spain

**Keywords:** Delphi, Patient-reported outcome measures, Quality of life, Systemic lupus erythematosus

## Abstract

**Background:**

Systemic lupus erythematosus (SLE) may result in great impact on patients’ quality of life, social relationships, and work productivity. The use of patient-reported outcome measures (PROMs) in routine care could help capture disease burden to guide SLE management and optimize disease control. We aimed to explore the current situation, appropriateness, and feasibility of PROMs to monitor patients with SLE in routine care, from healthcare professionals’ and patients’ perspectives.

**Methods:**

A scientific committee developed a Delphi questionnaire, based on a focus group with patients and a literature review, including 22 statements concerning: 1) Use of PROMs in routine care (*n* = 2); 2) PROMs in SLE management (*n* = 13); 3) Multidisciplinary management of patients with SLE (*n* = 4), and 4) Aspects on patient empowerment (*n* = 3). Statements included in Sects. 2–4 were assessed from three perspectives: current use, appropriateness, and feasibility (with currently available resources). For each statement, panellists specified their level of agreement using a 7-point Likert scale. A consensus was reached when ≥ 70% of the panellists agreed (6,7) or disagreed (1,2) on each statement.

**Results:**

Fifty-nine healthcare professionals and 16 patients with SLE participated in the Delphi-rounds. A consensus was reached on the value of PROMs to improve SLE management (83%) and the key role of healthcare professionals (77%) and the need for a digital tool connected to the electronic medical record (85%) to promote and facilitate PROMs collection. PROMs most frequently used in clinical practice are pain (56%), patient’s global assessment (44%) and fatigue (39%), all on visual analogue scales. Panellists agreed on the need to implement multidisciplinary consultation (79%), unify complementary tests (88%), incorporate pharmacists into the healthcare team (70%), and develop home medication dispensing and informed telepharmacy programmes (72%) to improve quality of care in patients with SLE. According to panellists, patient associations (82%) and nurses (80%) are critical to educate and train patients on PROMs to enhance patient empowerment.

**Conclusions:**

Although pain, fatigue, and global assessment were identified as the most feasible, PROMs are not widely used in routine care in Spain. The present Delphi consensus can provide a road map for their implementation being key for SLE management.

**Supplementary Information:**

The online version contains supplementary material available at 10.1186/s41927-024-00401-x.

## Introduction

Systemic lupus erythematosus (SLE) is a complex and heterogeneous autoimmune disease, both in its course and clinical presentation [1], affecting mostly young women, who represent 90% of the patients with this disease [2–4]. In Spain, the prevalence of SLE is 210 in 100,000 inhabitants according to the EPISER 2016 study [5].

The disease burden of SLE is considerable, mainly in terms of its negative impact on patients’ health-related quality of life (HRQoL), social relationships, and work productivity [6, 7]. Patients report fatigue and pain as the most prevalent and debilitating symptoms [2, 7, 8]. In fact, in a survey carried out in Spain (*n* = 1263 patients with SLE), muscle and joint pain, and fatigue were reported by 75% and 74% of patients, respectively, as the symptoms with the highest impact on their daily lives [6].

As in other rheumatic diseases with high impact on patients' life, it becomes critical to include the patients' perspective in order to obtain a comprehensive assessment of the disease [9, 10]. International organizations such as the European Alliance of Associations for Rheumatology (EULAR) [11] or Outcomes Measures in Rheumatology (OMERACT) [12] have carried out various initiatives to promote and facilitate the incorporation of the patient's perspective in disease management. One of these initiatives has been the freely available online EULAR outcome measures library, which includes validated instruments (indices, questionnaires, scales, and others) for the measurement of patient-reported outcome (PRO) variables [9, 11].

PRO is a measure of the status of the patient’s health condition that comes directly from the patient without the response being interpreted by a clinician or anyone else [13]. Therefore, the assessment of PROs during patient follow-up, using specific instruments (Patient Reported Outcomes Measures, PROMs) [14], provides information on the impact of the disease on the patient's HRQoL, functional and emotional status. Moreover, PROMs evaluate those symptoms that are perceived by the patient, such as fatigue and pain, among others. PROMs are useful for incorporating the patient's perspective in the disease decision-making process, which has been identified as one of the main challenges in the management of patients with SLE [7, 15]. Thus, using PROMs in clinical practice could contribute to a better understanding of SLE management and thereby optimize disease control [2, 9, 16].

PROMs are also useful to patients and improve their clinical experience, primarily by facilitating communication [17]. Therefore, the proper use of PROMs is an important conceptual issue and an opportunity to build bridges in the partnership between patients and physicians [6, 18]. This is particularly relevant considering the previously described discordance between doctors and patients in different rheumatic diseases, including SLE [19]. While patients often base their disease activity evaluation on symptoms such as pain, fatigue, and quality of life or the impact of disease on their daily activities, physicians consider other relevant aspects as markers of inflammation in laboratory tests or damage in imaging to evaluate disease activity. Recognizing and addressing these differences in disease evaluation allows the use of PROMs to enhance mutual understanding and improve the overall management of SLE, providing additional relevant information from the patient's perspective during routine care [18, 20].

In this study, we aimed to explore the current situation, appropriateness, and feasibility of using PROMs to monitor patients with SLE in routine care, from both healthcare professionals’ and patients’ perspectives in Spain. These results will enable us to establish expert recommendations to incorporate PROMs in the current management and empowerment of patients with SLE.

## Methods

### Scientific committee

The study was led by a scientific committee of seven healthcare professionals: five rheumatologists (IC, MG, AM, MJC, TS), one hospital pharmacist (JB) and one nurse (LC).

Members of the scientific committee were selected based on their experience in managing patients with SLE.

### Delphi methodology

The Delphi technique is a widely used consensus method used in research to achieve a consensus on a particular topic, preserving participants’ anonymity. It is conducted over consecutive survey rounds (usually two) and answered by a panel of participants with relevant expertise in the research field [21]. During Delphi rounds, panellists were asked to rate different statements or questions, providing controlled feedback on the previous round’s group results [22]. Participants may then adjust their initial ratings based on feedback from the overall group in several subsequent iterations [23].

In this study, a two-round Delphi was performed; the first-round questionnaire was available from 26th July 2022 to 23rd September 2022, while the second round took place from 19th October 2022 to 7th November 2022. Each round consisted of an electronic questionnaire which required no more than 20 min to complete.

### Delphi questionnaire

The scientific committee developed the Delphi questionnaire based on a focus group comprising patients with SLE in addition to a comprehensive literature review on PROMs in lupus.

The focus group is a variant of a group interview in which participants describe their perceptions, opinions, beliefs, and attitudes towards a specific topic [24]. An online focus group with patients with SLE [*n* = 8 patients with SLE and *n* = 1 caregiver, 78% women, mean age 46.4 (SD: 14.48) years; mean time from diagnosis 26.3 (SD:14.51)] was conducted to explore the current management of SLE in routine clinical practice and to identify related challenges and unmet needs from the patients’ perspective. Patients with SLE were contacted and invited to participate in the focus group by the National Spanish patient advocacy group (Federación Española de Lupus, FELUPUS).

Subsequently, a literature review was conducted in May 2022 to synthesise the available evidence for the current challenges and unmet needs highlighted by the patient focus group and to identify and characterise available PROMS used in SLE management. For that purpose, international databases (PubMed) and grey literature (www.eular.org; www.ser.es; https://omeract.org; www.felupus.org) were searched. Details of the search strategy conducted can be found in Supplementary File S1.

After the literature review, the multidisciplinary committee assessed the PROMs used in SLE management and agreed to prioritise the most essential and feasible in clinical practice. In addition, for the selection process, the validation and measurement properties of the PROMs were considered, including domains covered, the total number of items, feasibility with the time required to complete and whether any training was required, construct validity and sensitivity to change. The selected PROMs were then presented to the panellists in two Delphi rounds.

The literature review identified 31 PROMs related to SLE management. Nevertheless, based on the criteria of appropriateness and feasibility in clinical practice of the scientific committee in the Delphi questionnaire, a total of 13 PROMs were included.

Two versions of the electronic questionnaire were developed, one for healthcare professionals (Supplementary File S2) and another for patients with SLE (Supplementary File S3), to make it easier for the latter to understand the wording.

The Delphi questionnaire employed in the first round targeting healthcare professionals included 22 statements covering four main sections: 1) Use of PROMs in clinical practice (*n* = 2); 2) PROMs in SLE management (*n* = 13): Visual Analogic Scale ([VAS]-Pain, VAS-Fatigue, Patient's global assessment [PGA] of the disease), EuroQoL-5 Dimensions (EQ-5D), Lupus Impact Tracker (LIT), Hospital Anxiety and Depression Scale (HADS), Functional Assessment of Chronic Illness Therapy Fatigue (FACIT-Fatigue), Fatigue Severity Scale (FSS), Work Productivity and Activity Impairment: Lupus (WPAI: Lupus), Oviedo Sleep Questionnaire (OSQ), Pittsburgh Quality of Sleep Inventory (PSQI), Treatment Satisfaction Questionnaire for Medication (TSQM), Lupus Damage Index Questionnaire (LDIQ), LupusPRO, Multi-Dimensional Health Assessment Questionnaire (MDHAQ); 3) Multidisciplinary management of patients with SLE (*n* = 4); 4) Patient empowerment (*n* = 3).

Statements encompassed in Sects. “PROMs in SLE management”, “Use of PROMs in clinical practice” and “Multidisciplinary management of patients with SLE” were assessed from three perspectives: current use, appropriateness, and feasibility (to implement the statement with currently available resources you have at your disposal).

The Delphi questionnaire addressed specifically to patients with SLE included seven statements covering the same four sections listed above.

For each statement, panellists specified their level of agreement using a 7-point Likert scale (1, strongly disagree; 2, mostly disagree; 3, somewhat disagree; 4, neutral; 5, somewhat agree; 6, mostly agree; 7, strongly agree).

The questionnaire employed in the second round covered those statements that did not reach consensus in the first round, and the statements related to those PROMs that reached 50–70% agreement were taken to the second round.

### Panellists

Delphi questionnaire was addressed to healthcare professionals involved in SLE management, including rheumatologists, specialists in internal medicine, nephrologists, hospital pharmacists, nurses, dermatologists, and psychologists, as well as to patients with SLE. Healthcare professionals were selected and invited to participate by the study sponsor, based on their experience in managing patients with SLE. The Spanish National patient advocacy group FELUPUS contacted and invited patients to participate in the Delphi rounds. There is no ideal number of participants for a focus group neither for Delphi panellists. However, it is estimated that an adequate number for focus group is between 4 and 12 [25] and from 6 to 50 for Delphi consultation [22]; the decision is empirical and pragmatic, the qualities of the panel of experts being more important than their number [26]. Based on this, 59 physicians and 16 patients were proposed to participate.

This study did not involve any collection of drug-related data and does not meet any of the criteria required to be considered as an observational clinical trial as defined in Article 2.1.i) in Spain’s Royal Decree 1090/2015 of December 4th as provided by the Ministerio de la Presidencia, Relaciones Con Las Cortes Y Memoria Democrática, which regulates observational studies with medicines for human use [27] or as defined by the Spanish Agency for Medicines and Health Products [28]. Therefore, consistent with the Royal Decree and the Spanish Agency for Medicines and Health Products, evaluation and approval by an ethics committee, or IRB approval, was not required for this study [27, 28]. Prior to beginning the questionnaire, all participants were informed of the study structure, scientific committee, and the confidentiality and data protection measures that were in place. Written informed consent was obtained by all participants by checking a box indicating that they read and accepted the survey and agreed to participate in the study with all the conditions described.

### Consensus definition

A statement reached consensus if at least 70% of the panellists either mostly agreed / strongly agreed (ratings of 6–7) or mostly disagreed / strongly disagreed (ratings of 1–2).

### Data analysis

The descriptive analysis of panellist characteristics and the percentage of participants who selected each option was conducted using STATA statistical software, V.14. The percentages described in the text refer to the final scores (score of the round in which consensus was achieved).

## Results

### Panellists’ characteristics

A total of 75 panellists, comprising 79% healthcare professionals and 21% patients, participated in the first Delphi round, with a response rate in the second round of 97% among healthcare professionals and 94% among patients. The healthcare professionals represented diverse medical specialities such as rheumatologists (51%), specialists in internal medicine (17%), nephrologists (9%), hospital pharmacists (9%), nurses (9%), dermatologists (3%), and psychologists 3%), all of them with solid experience in SLE management and broad geographic representation (Table 1).

**Table 1:** Main panellists’ characteristics

	**Healthcare professionals**	**Patients**
**Sex, n (%)**		
Men	30 (50.85)	4 (25.0)
Women	29 (49.15)	12 (75.0)
**Age, mean (SD)**	49.97 (10.00)	44.19 (15.43)
**Years from diagnosis, mean (SD)**	-	15.19 (12.03)
**Years from diagnosis, median**		10
**Experience, n (%)**		
Junior faculty (< 15 years)	14 (23.73)	-
Intermediate (15–30 years)	29 (49.15)	
Senior faculty (> 30 years)	16 (27.12)	
**Years of experience with SLE patients, mean (SD)**	18.34 (9.00)	-
**Medical specialities, %**	50.8	
Rheumatologists	16.9	
Internal medicine Nephrologists	8.5	
Hospital pharmacists	8.5	
Nurses	8.5	
Dermatologists	3.4	
Psychologists	3.4	

*SD* Standard deviation, *SLE* systemic lupus erythematosus

### Use of PROMs in SLE management

**Fig. 1:**
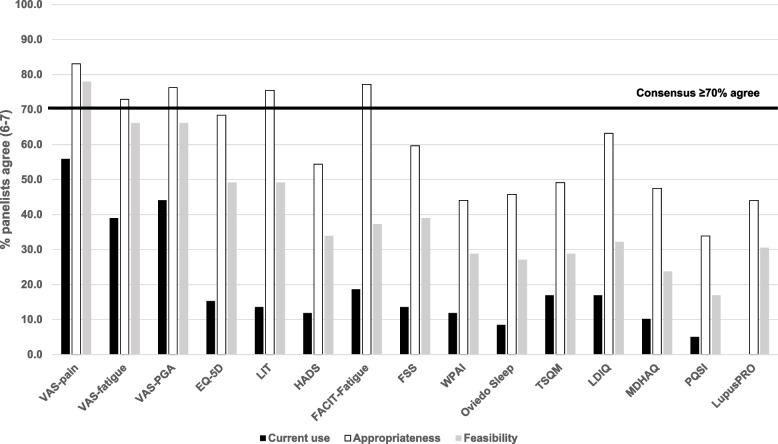
Current use, appropriateness and feasibility of PROMs used in clinical practice

The PROMs most frequently used in clinical practice by the panellists are VAS-Pain (56%), VAS-PGA (44%) and VAS-Fatigue (39%). A consensus was reached regarding the appropriateness of VAS-pain, VAS-PGA, VAS-fatigue, FACIT-Fatigue, and LIT. However, only VAS-Pain was considered feasible to use in clinical practice (78%). All PROMs evaluated for current use, appropriateness and feasibility are presented in Fig. 1 and Table 2. Although VAS-Pain, VAS-Fatigue, VAS-PGA, LIT, and FACIT were considered appropriate instruments (> 70% agreement), none of them were considered feasible with the actual resources.

**Table 2:** Statements included in the Delphi questionnaire and agreement reached among panellists. Consensus recommendations are highlighted in bold

	Current use			Appropriateness			Feasibility		
Statement	D (%)	N (%)	A (%)	D (%)	N (%)	A (%)	D (%)	N (%)	A (%)
**PROMs in SLE management**									
Visual Analog Scale (VAS 0-100 mm) could be used to evaluate pain, fatigue, and general condition of the patient with SLE. Next, we ask you to assess the use of VAS in each of these three aspects of the disease:									
VAS Pain	11.86	32.20	55.93	3.39	13.56	83.05	3.39	18.64	77.97
VAS Fatigue	18.64	42.37	38.98	1.69	25.42	72.88	5.09	28.81	66.10
VAS PGA	13.56	42.37	44.07	0.00	23.73	76.27	6.78	27.12	66.10
Generic quality of life Questionnaire EuroQoL-5 Dimensions (EQ-5D) to assess pain, quality of life, functionality, and emotional state of the patient with SLE	33.90	50.85	15.25	5.26	26.32	68.42	15.25	35.59	49.15
Lupus Impact Tracker (LIT) to evaluate the quality of life of the patient with SLE ^b^	42.37	44.07	13.56	1.75 ^b^	22.81 ^b^	75.44 ^b^	16.95	33.90	49.15
Hospital Anxiety and Depression Scale (HADS) to assess the emotional state of the patient with SLE ^b^	40.68	47.46	11.86	1.75 ^b^	43.86 ^b^	54.39 ^b^	22.03	44.07	33.90
Functional Assessment of Chronic Illness Therapy – Fatigue (FACIT-Fatigue) to assess the fatigue of the patient with SLE	40.68	40.68	18.64	1.75 ^b^	21.05 ^b^	77.19 ^b^	18.64	44.07	37.29
Fatigue Severity Scale (FSS) to assess the fatigue of the patient with SLE	50.85	35.59	13.56	1.75 ^b^	38.6 ^b^	59.65 ^b^	18.64	42.37	38.98
Work Productivity and Activity Impairment: Lupus (WPAI: Lupus) to assess work productivity in the patient with SLE	59.32	28.81	11.86	6.78	49.15	44.07	23.73 ^b^	47.46	28.81
Oviedo sleep questionnaire to assess sleep disturbances in the patient with SLE	55.93	35.59	8.47	3.39	50.85	45.76	25.42	47.46	27.12
Treatment Satisfaction Questionnaire for Medication (TSQM version 1.4) to assess the satisfaction of the patient with SLE with the treatment	55.93	27.12	16.95	3.39	47.46	49.15	23.73	47.46	28.81
Lupus Damage Index Questionnaire (LDIQ) to assess irreversible damage in the patient with SLE	47.46	35.59	16.95	7.02 ^b^	29.82 ^b^	63.16 ^b^	23.73	44.07	32.20
Multi-Dimensional Health Assessment Questionnaire (MDHAQ) (R808-NP2-Spanish) to assess the functional capacity, pain, fatigue, disease activity and emotional state of the patient with SLE	55.93	33.90	10.17	5.08	47.46	47.46	32.20	44.07	23.73
Pittsburgh Quality of Sleep Inventory (PQSI) Questionnaire to assess sleep disturbances in the patient with SLE	57.63	37.29	5.08	15.25	50.85	33.90	37.29	45.76	16.95
LupusPRO to assess the functional capacity, pain, quality of life, emotional state, and cognitive alterations in the patient with SLE	54.24	37.29	8.47	5.08	50.85	44.07	35.59	33.90	30.51
**Use of PROMs in clinical practice**									
Incorporating the patient’s perspective using PROMs in clinical practice contributes to improving patients with SLE management^**a**^	5.33	12.00	82.67	NA					
The following strategies contribute to promoting the use of PROMs in clinical practice^**a**^:	NA								
Assigning a healthcare professional, in addition to the physician, to support the patient in completing the PROMs may promote the use of PROMs in clinical practice	5.33	17.33	77.33						
Having a digital tool to collect PROMs connected to the electronic medical record may promote the use of PROMs in clinical practice	2.67	12.00	85.33						
**Multidisciplinary management of patients with SLE**									
Multidisciplinary consultation optimises the management of SLE by facilitating and streamlining the interaction/coordination between specialists^**a**^	17.33	32.00	50.67	1.33	20.00	78.67	5.33	38.67	56.00
The unification of complementary tests facilitates and improves patient care (reducing duplications for the clinician and patient)^**a**^	12.00	24.00	64.00	0.00	12.00	88.00	1.33	30.67	68.00
Incorporating hospital pharmacists into the healthcare team would improve medication management in patients with SLE (e.g., to avoid possible drug interactions)	18.64	42.37	38.98	7.02 ^b^	22.81 ^b^	70.18 ^b^	10.17	37.29	52.54
Telematic medication dispensing programmes (telepharmacy) optimise medication management in outpatient patients with SLE by reducing the need to travel to the hospital pharmacy for medication dispensation and monitoring^**a**^	22.67	30.67	46.67	1.33	26.67	72.00	1.33	41.33	57.33
**Patient empowerment**									
Patient associations could play an important role in informing and training patients in the use of PRO/PROMs in clinical practice^**a**^	10.67	46.67	42.67	4.17 ^b^	13.89^b^	81.94^b^	4.00	41.33	54.67
In addition to the physician responsible for the patient follow-up, nursing plays a key role in training and educating the patient in the use of PRO/PROMs	11.86	32.20	55.93	3.39	16.95	79.66	3.39	30.51	66.10
PROMs can facilitate patient participation in decision-making and thus improve adherence to treatment^**a**^	13.33	44.00	42.67	2.67	25.33	72.00	5.33	40.00	54.67

*A* Agreement, *D* Disagreement, *N* Neutral, *PRO* Patient-reported outcome, *PROM* Patient-reported outcome measure, *SLE* Systemic lupus erythematosus

^a^Statements addressed to experts and patients

^b^Statements included in the second round

### Use of PROMs in clinical practice

Panellists agreed (83%) that incorporating the patient perspective using PROMs in clinical practice contributes to improving the management of patients with SLE. Additionally, they reached a consensus that the use of PROMs in clinical practice could be promoted by assigning a healthcare professional to support the patient in completing the PROMs (77%) and a digital tool to collect PROMs connected to the electronic medical record (85%) (Table 2).

### Multidisciplinary management of patients with SLE

The multidisciplinary management of SLE patients is tailored to each individual in clinical practice, requiring the expertise of rheumatologists and internists (reference specialists). Depending on the patient's specific condition, nephrologists and dermatologists may also be required. Additionally, the involvement of other healthcare professionals, such as hospital pharmacists and specialized nurses, is essential for the ongoing follow-up and comprehensive care of the patient.Panellists considered it appropriate (79%) to implement multidisciplinary consultation to optimise the management of SLE by facilitating and streamlining the interaction/coordination between specialists. However, a consensus was not reached regarding its feasibility in clinical practice with the actual resources (Table 2).

Likewise, panellists agreed (88%) to unify complementary tests to facilitate and improve the quality of patient care (reducing duplications for the clinician and patient). Although this procedure seems widespread in clinical practice, there was no consensus on its feasibility (Table 2).

Incorporating hospital pharmacists into the healthcare team to facilitate medication management in SLE patients (e.g., to avoid possible drug interactions) was considered appropriate (70%). However, the panellists indicated that it did not reflect current clinical practice and was not considered feasible (Table 2).

Panellists considered the development of telematic medication dispensing programmes appropriate (72%) to optimise medication management in outpatient patients with SLE by reducing the need to travel to the hospital pharmacy for drug dispensation and monitoring, despite the fact that current implementation and feasibility are hindered by the currently available resources (Table 2).

### Patient empowerment

All strategies proposed to enhance patient empowerment were considered appropriate; the panellists agreed that patient associations (82%) and nurses (80%) could inform and train patients to use PROMs, thus facilitating patient participation in decision-making and improving adherence to treatment (72%). However, none of these strategies were currently implemented in clinical practice nor considered feasible (Table 2).

## Discussion

Our results provide key information on the current use of PROMs to monitor patients with SLE in routine clinical practice in Spain from both patients’ and healthcare professionals’ perspective. In this context, the information gathered in the Delphi consultation enables us to draw conclusions to improve the current situation concerning the use of PROMs and to define strategies to promote a holistic and integrated approach to SLE management.

Patients’ perceptions of their symptoms, function and HRQoL are increasingly recognized as outcome measures. These measures complement traditional provider-collected datain clinical practice, particularly for evaluating rheumatic diseases like SLE [9]. Despite their value, current methods for recording PROs have notable limitations.. The development of PROMs and the adoption of enabling technologies (smartphones, tablets, internet access, and electronic health record integration) can facilitate their routine use [29]. Moreover, digital PROMs (ePROMs) may be easier to standardize, thus achieving greater collection consistency, frequency, and accuracy [30]. To be useful, ePROMs must be user-friendly, require minimal action from patients or providers, and integrate seamlessly into daily routines [30]. In addition, healthcare professionals support the use of digital tools to collect PROMs linked to electronic medical records, enhancing their clinical application [31]. In this context, it is important to note that patients with SLE who completed PROMs had a lower probability of relapse and a higher likelihood of achieving remission [32] as well as an enhanced clinical experience, primarily due to better communication with the healthcare provider [17].

Several PROMs are available for SLE [9, 33]. The Delphi consultation confirm that PROMs are not widely used in routine care, with only VAS-pain, VAS-PGA, and VAS-fatigue reported over 40% of panellists. This results alint with the European INTEGRATE project, which also found PROMs are rarely used in routine clinical practice [34].

Although panellists considered the use of PROMs during patient management to be appropriate for assessing pain, fatigue and HRQoL, a consensus was not reached regarding their feasibility with the actual resources. For patients with SLE, incorporating PROMs in routine care can be particularly cost-effective due to the complex and heterogeneous nature of the disease. Selecting feasible PROMs can lead to better symptom evaluation and management, patient-centred treatments, and, ultimately, better use of resources, lowering healthcare costs by preventing severe flares and hospitalizations. Main barriers and challenges related to implementation and data integration are developing appropriate tools, training staff, integrating PROMs into electronic health record (EHR) systems to be used in clinical workflows, and ensuring that it informs treatment decisions.

Most physicians consider a lack of feasibility because the actual scenario in routine care does not include this infrastructure (appropriate tools in EHR and expert nurses) to incorporate PROMs in the workflow. In particular, panellists agreed on the appropriateness of VAS-Pain, VAS-PGA, VAS-Fatigue and FACIT-fatigue; however, only VAS-Pain was considered feasible in routine care. It is surprising since VAS is the same tool, and one would expect similar feasibility of all VAS. This discrepancy may be because panellists were assessing the use of all three VAS (VAS-Pain, VAS-PGA and VAS-Fatigue) during the consultation, considering it unfeasible and, therefore, prioritising the use of VAS-Pain.

The assessment of disease impact is key in the management of SLE. Panellists agreed on considering LIT, a unidimensional, 10-question instrument validated among adults and children with SLE [35], as the most appropriate PROM for that purpose. It takes less than three minutes to complete and is not considered disruptive or burdensome by patients and physicians [33]. Most physicians and patients have reported that the LIT helped them discuss the impact of SLE on patients’ lives and improved communication between them [36]. Of note, among PROMs considered appropriate by the panellists in our study, LIT is the only one specific for patients with SLE.

Depression and anxiety, common emotional impacts of SLE, significantly affect patients' lives [33]. However, according to our results, depression and anxiety are not currently assessed in routine care. Patients with self-reported symptoms of anxiety and depression reported a worse HRQoL, greater fatigue levels and a higher disease burden on their daily lives [37]; therefore, early recognition of depression and anxiety in patients with SLE would seem crucial.

These results highlight the need for medical education and resources to promote the use of PROMs in clinical practice. Further studies needed to assess the impact of the use of PROMs on disease activity, progression, and healthcare resource use during patient follow-up.

Therefore, the strategies to promote a holistic and integrated approach, as drawn from our study, are particularly relevant. A multidisciplinary approach to improve specialists’ interaction and coordination is crucial. SLE is a multiorgan disease; although fever, rash and arthritis are the classic initial symptoms, abrupt onset with target organ involvement is also common. A wide range of clinical manifestations, including haematological, renal, neuropsychiatric, ocular, or mucocutaneous involvement, can be presented in SLE patients [38].

Thus, a multidisciplinary team appears optimal since different clinical manifestations need to be addressed by multiple specialists and the panellists agreed on this aspect. Particularly, panellists agreed on enhancing the hospital pharmacist's role in medication management, including adherence monitoring, defining discontinuation criteria, and identifying medication-related problems. They also supported home medication dispensing and telepharmacy programs to optimize patient care and reduce travel. In this context, several initiatives have promoted telemedicine implementation [39, 40].

Incorporating patients' perspectives in SLE management paves the way towards patient-centred care. Patient education is an important aspect of patient engagement since knowledge is the first and necessary step for self-management [41]. Delphi results highlighted the nurse's role in patient education and training with PROMs. Nurses have a long tradition in counselling, teaching self-care,, providing emotional support, and skills training [41]. The RECOMIENDAles, in Spain highlighted the importance of nurses in multidisciplinary teams for patient education and monitoring, among others [42]. Also in Spain, a recent survey, found that 40% of SLE patients reported scarce physician–patient dialogue, with most receiving informative material during visits [6]. Therefore, nurses, alongside physicians are crucial in promoting patient information. Patient advocacy and support programs can also enhance patient education though awarenes campaigns and support programs, improving adherence and clinical and humanistic outcomes [7, 43].

Finally, given the chronic, multisystemic, heterogeneous nature of the disease, PROMs are essential to assess disease activity and HRQoL in patients with SLE [44, 45]. The incorporation of the patient’s perspective into shared decision-making processes may improve disease control in an early stage, avoiding irreversible organ damage progression.

The Spanish Public Healthcare System (Sistema Nacional de Salud, SNS) provides comprehensive care for lupus patients. This includes access to specialists, medications, and hospital services with minimal out-of-pocket costs for residents. Typical care for lupus patients involves a multidisciplinary approach due to the complexity and systemic nature of the disease, with management by various healthcare professionals to address its wide range of symptoms and complications. The initial patient journey often starts with a visit to a primary care physician, who evaluates symptoms and request preliminary blood tests. If lupus is suspected, the primary care physician will refer the patient to a rheumatologist. However, as symptoms may be non-specific, some patients may be referred by other specialists. In general, rheumatologists are the primary specialists managing lupus, responsible for diagnosis, medication prescription, disease monitoring, and treatment adjustments. A multidisciplinary team, such as dermatologists and nephrologists, supports comprehensive care. Regular monitoring at rheumatology clinics in both academic and non-academic centres include evaluating disease activity, assessing comorbidities, and identifying potential medication side effects. In Spain, only six centres are specialized in the management of systemic autoimmune diseases: (CSUR): three in Madrid (HU La Paz, HU Gregorio Marañón, HU 12 de Octubre), two in Catalonia (HU Vall D'Hebron and Hospital Clinic de Barcelona) and one in Cantabria (HU Marqués de Valdecilla). These centres, integrated into the Spanish National Health System, provide specialized care and ensure that SLE patients receive appropriate diagnosis and treatment.

Our study has several limitations. First, study participants were limited to Spanish healthcare professionals, so extrapolating the findings to other settings should be done with caution. Second, the recommendations reflect the opinion of a multidisciplinary panel. Although no significant differences are expected, different participants could have reached different recommendations. Finally, these recommendations are based on current therapeutic strategies; therefore, the development of new strategies that may change the disease burden could affect these recommendations, and periodic updates would be required.

## Conclusion

In conclusion, strategies to promote a holistic and integrated approach to managing SLE include the creation of multidisciplinary teams and incorporating patients’ perspectives through PROMs, providing a unique insight into the patient’s perception of SLE disease activity.

In this study, we agreed on the most appropriate PROMs for the SLE in clinical practice since the use of PROMs can facilitate patient participation in decision making and thus improve adherence to treatment. Although PROMs are not widely used in routine care in Spain, healthcare professionals are aware of the need to incorporate them in the care of patients with SLE and agreed on the need for patient education and providing additional resources to promote their implementation. The results of the present Delphi consensus can be the road map for strategies to implement PROMs in routine care and to develop studies to evaluate the impact of PROMs collection on management of patients with SLE.

## Supplementary Information

Supplementary Material 1.

Supplementary Material 2.

Supplementary Material 3.

## Data Availability

The datasets used and/or analysed during the current study are available from the corresponding author on reasonable request.

## Abbreviations

ePROMs: Digital PROMs
FACIT: Functional assessment of chronic illness therapy fatigue
FSS: Fatigue severity scale
HADS: Hospital anxiety and depression scale
HRQoL: Health-related quality of life
LDIQ: Lupus damage index questionnaire
LIT: Lupus impact tracker
MDHAQ: Multi-dimensional health assessment questionnaire
OSQ: Oviedo sleep questionnaire
PGA: Patient's global assessment
PROMs: Patient-reported outcome measures
PSQI: Pittsburgh quality of sleep inventory
SLE: Systemic lupus erythematosus
TSQM: Treatment satisfaction questionnaire for medication
VAS: Visual analogic scale
WPAI: Work productivity and activity impairment
