# Correction: Patient-reported outcome measures for systemic lupus erythematosus: an expert Delphi consensus to guide implementation in routine care

**DOI:** 10.1186/s41927-024-00432-4

**Published:** 2024-10-25

**Authors:** Isabel Castrejón, Laura Cano, María José Cuadrado, Joaquín Borrás, Maria Galindo, Tarek C. Salman-Monte, Carlos Amorós, Carmen San Román, Isabel Cabezas, Marta Comellas, Alejandro Muñoz

**Affiliations:** 1 Departamento de Medicina, Servicio de Reumatología, Hospital General Universitario Gregorio MarañónInstituto de Investigación Sanitaria Gregorio MarañónUniversidad Complutense de Madrid, C. del Dr. Esquerdo, 46, Madrid, 28007 Spain; 2 Enfermería Reumatología, H. Regional de Málaga, Málaga, Spain; 3 Servicio de Reumatología, H. Clínica Universitaria de Navarra, Madrid, Spain; 4 Farmacia Hospitalaria. H. de Sagunto, Valencia, Spain; 5 Servicio de Reumatología, H. 12 de Octubre, Madrid, Spain; 6 Servicio de Reumatología, H. del Mar, Barcelona, Spain; 7 GlaxoSmithKline, Madrid, Spain; 8 Outcomes’10, Castellón de La Plana, Spain; 9 Servicio de Reumatología, H. Universitario Virgen del Rocío, Sevilla, Spain

**Correction:** ***BMC Rheumatol*** **8** **, 31 (2024).**

10.1186/s41927-024-00401-x

Following publication of the original article [1], the authors reported an error in the Acknowledgements section where “A. Martin (H. Lozano Blesa)” should be changed to “A. Marín (H. Lozano Blesa)”.

The originally published Acknowledgements section was:

The authors would like to thank to all participants in the Delphi Rounds, without them this project would not have been possible: E. Morales (H. 12 de Octubre), A. Martín (H. de Poniente), A. Ávila (H. Dr Peset), M. Uriol (H. Son Espases), L. Quintana (H. Clinic), E. Ramírez (H. La Princesa), P. Lopez (H. General de Tomelloso), T. Palanques (H. Joan XXIII de Tarragona), A. Gilabert (H. Granollers), E. Chavarría (H. Universitario Infanta Leonor), C. Mouriño (H. Meixoeiro), C. Martin (H. Candelaria), S. Garcia (H. Moises Broggi), P. Garcia (H. Ramon y Cajal), M. Ferreiro (H. Las Cruces), R. Serrano (H. Universitari Germans Trias i Pujol), C. Domínguez (H. Macarena), N. Ortego (H. PTS), J. Callejas (H. PTS), G. Espinosa (H. Clinic), L. Pallarés (H. Son Espases), A. Robles (H. La Paz), D. Bellido (H. Ciudad Real), J. Barbado (H. Río Hortega), C. Romero (H. regional de Málaga), A. Martin (H. Lozano Blesa), I. Rodriguez (Mutua de Terrasa), I. Altabás (H. Meixoeiro), C. Pego (H. Meixoeiro), A. Aragón (H. de Getafe), E. Trujillo (H. Insular), I. Rua (H. Dr Negrín), M. Garcia (H. Ramon y Cajal), A. Fernádez (H. regional de Málaga), J. Calvo (H. Universitario Alava), C. Marras (H. Arrixaca), A. Garcia (H. Virgen de la Salud), V. Torrente (H. Villafranca Penedés), C. Moriano (H. Léon), M. Aguirre (H. de Córdoba), M. Freire (Complejo H. Universitario de La Coruña), R. Blanco (H. de Valdecilla), L. Riancho (H. Sierrallana), J. Martínez (H. Gregorio Marañón), P. Navarro (H. Elche), T. Cobo (H. Infanta Sofia), (A) Domínguez (NA), C. Valdés (NA), J. Antón (H. Sant Joan de Déu), J. Andreu (H. Puerta de Hierro), O. Sánchez (FJD), (B) Tejera (H. Insular), G. Mercadal (H. Mateu Orfila). Additionally, we would like to thank the Spanish patients’ advocacy groups FELUPUS for its support during Project development.

Medical writing was provided by Victor Latorre, PhD, at Outcomes’10, Spain, and was funded by GSK.

The corrected Acknowledgements section should read:

The authors would like to thank to all participants in the Delphi Rounds, without them this project would not have been possible: E. Morales (H. 12 de Octubre), A. Martín (H. de Poniente), A. Ávila (H. Dr Peset), M. Uriol (H. Son Espases), L. Quintana (H. Clinic), E. Ramírez (H. La Princesa), P. Lopez (H. General de Tomelloso), T. Palanques (H. Joan XXIII de Tarragona), A. Gilabert (H. Granollers), E. Chavarría (H. Universitario Infanta Leonor), C. Mouriño (H. Meixoeiro), C. Martin (H. Candelaria), S. Garcia (H. Moises Broggi), P. Garcia (H. Ramon y Cajal), M. Ferreiro (H. Las Cruces), R. Serrano (H. Universitari Germans Trias i Pujol), C. Domínguez (H. Macarena), N. Ortego (H. PTS), J. Callejas (H. PTS), G. Espinosa (H. Clinic), L. Pallarés (H. Son Espases), A. Robles (H. La Paz), D. Bellido (H. Ciudad Real), J. Barbado (H. Río Hortega), C. Romero (H. regional de Málaga), A. Marín (H. Lozano Blesa), I. Rodriguez (Mutua de Terrasa), I. Altabás (H. Meixoeiro), C. Pego (H. Meixoeiro), A. Aragón (H. de Getafe), E. Trujillo (H. Insular), I. Rua (H. Dr Negrín), M. Garcia (H. Ramon y Cajal), A. Fernádez (H. regional de Málaga), J. Calvo (H. Universitario Alava), C. Marras (H. Arrixaca), A. Garcia (H. Virgen de la Salud), V. Torrente (H. Villafranca Penedés), C. Moriano (H. Léon), M. Aguirre (H. de Córdoba), M. Freire (Complejo H. Universitario de La Coruña), R. Blanco (H. de Valdecilla), L. Riancho (H. Sierrallana), J. Martínez (H. Gregorio Marañón), P. Navarro (H. Elche), T. Cobo (H. Infanta Sofia), (A) Domínguez (NA), C. Valdés (NA), J. Antón (H. Sant Joan de Déu), J. Andreu (H. Puerta de Hierro), O. Sánchez (FJD), (B) Tejera (H. Insular), G. Mercadal (H. Mateu Orfila). Additionally, we would like to thank the Spanish patients’ advocacy groups FELUPUS for its support during Project development.

Medical writing was provided by Victor Latorre, PhD, at Outcomes’10, Spain, and was funded by GSK.

The original article [1] has been updated.
