# Supplementary material for: Patient-reported outcome measures for systemic lupus erythematosus: an expert Delphi consensus to guide implementation in routine care
Source: BMC Rheumatol. 2024 Jul 16;8:31. doi: 10.1186/s41927-024-00401-x (PMC11251319; doi:10.1186/s41927-024-00401-x)
Supplement: Supplementary file 1 — Supplementary Material 1. [file 41927_2024_401_MOESM1_ESM.docx]

Supplementary Table S1. Search strategy conducted in PubMed

| **Search 1** | "systemic lupus erythematosus" AND ("Quality of Life"[Mesh] OR Health-Related Quality Of Life[tiab] OR Patient reported outcome*[tiab] OR Patient related outcome*[tiab] OR Patient-reported outcome*[tiab] OR Patient-related outcome*[tiab] OR Patient reported outcome*[ot] OR Patient related outcome*[ot] OR Patient-reported outcome*[ot] OR Patient-related outcome*[ot] OR "Patient Satisfaction"[Mesh] OR "Patient Preference"[Mesh] OR"unmet needs"[tiab]) |
| --- | --- |
| **Search 2** | ("lupus" OR "SLE" OR "systemic lupus erythematosus") AND ("multidisciplinary unit" OR "multidisciplinary handling" OR "multidisciplinary care“ OR “multi-professional”) |
