# Supplementary material for: Patient-reported outcome measures for systemic lupus erythematosus: an expert Delphi consensus to guide implementation in routine care
Source: BMC Rheumatol. 2024 Jul 16;8:31. doi: 10.1186/s41927-024-00401-x (PMC11251319; doi:10.1186/s41927-024-00401-x)
Supplement: Supplementary file 2 — Supplementary Material 2. [file 41927_2024_401_MOESM2_ESM.pdf]

Supplementary File 2: Delphi expert questionnaire focused on the current management of the patient living with systemic lupus erythematosus and the current challenges in clinical practice.

INFORMATION SHEET

You have been invited to participate in the project **"Current management of patients living with systemic lupus erythematosus. Current Challenges in Clinical Practice".**

This project is advised by the scientific committee formed by:

Name	Service / Hospital
**Isabel Castrejón (Project Coordinator)**	Rheumatology Department. Hospital Gregorio Marañón (Madrid)
**Maria Galindo**	Rheumatology Department. Hospital 12 de octubre (Madrid)
**Alejandro Muñoz**	Rheumatology Department. Hospital Virgen del Rocío (Sevilla)
**María Jose Cuadrado**	Rheumatology Department. Hospital Clínica Universitaria de Navarra (Madrid)
**Tarek Salman**	Rheumatology Department. Hospital del Mar (Barcelona)
**Joaquín Borrás**	Hospital Pharmacy Service. Hospital de Sagunto (Valencia)
**Laura Cano**	Nursing Rheumatology. Hospital Regional de Málaga (Málaga)

WHAT IS THE GOAL OF THE PROJECT?

The objective of the project is to agree and propose strategies to promote the implementation of patient-reported variables (PRO) and their measurement instruments (PROM) in the follow-up of patients with systemic lupus erythematosus in clinical practice.

WHAT DOES YOUR PARTICIPATION INVOLVE?

Your participation will consist of responding to **two rounds of Delphi** consultation through a **questionnaire in electronic format**, the completion of which will require a **maximum of 25-30 minutes**.

The Delphi **consensus first round questionnaire** presents a series of questions written as statements, where you must indicate **your degree of agreement, based on your experience** in clinical practice on a 7-point Likert scale where 1= "strongly disagree" and 7= "strongly agree".

For each statement, you will need to consider three perspectives:

- Current situation (if it is currently being carried out in your environment)
- Adequacy (if you consider it appropriate/relevant to carry it out in clinical practice)
- Feasibility (if you consider is possible to implement it with the resources at your disposal)

At the end of the questionnaire, you will have a free text section where you can make the comments you consider appropriate or clarify the aspects you consider relevant. In addition, you will be able to indicate aspects that are important to you and not included in the Delphi questionnaire, which may be incorporated into the second round if the scientific committee deems it appropriate.

In the **second round of Delphi consultation**, only those statements that do not reach consensus in the first round or aspects proposed by the participants and not considered in the first round will be presented. The objective of this second round is to reach consensus on the answers and, therefore, the second questionnaire will be personalized for each participant, including the overall and individual score obtained in the first round.

CONFIDENTIALITY

Data protection

In compliance with data protection regulations, in particular Regulation (EU) 2016/679, of the European Parliament and of the Council, of 27 April 2016, we inform you that the personal data you provide us through this survey will be incorporated into the files and systems under the responsibility of "Glaxosmithkline SA (GSK) CIF-A28228526" will be used for communications related to the study "Current Management of the Patient Living with Systemic Lupus Erythematosus. Current Challenges in Clinical Practice".

Your data has been transferred to OUTCOMES'10 in order to carry out the study, as well as the necessary communications during the process of carrying it out, being deleted after the publication of the manuscript, within the legal deadlines established for this purpose according to current legislation. Outcomes'10 guarantees the adoption of the necessary technical and organisational measures to ensure the confidential treatment of personal data. There are no plans to transfer data. We inform you that international transfers are not made outside the European Economic Area. And that this processing does not imply the existence of automated decision-making or profiling.

Categories of Personal Data: Researchers

The basis for legitimising the processing is the consent of the data subject (Article 6(1)(a) GDPR).

For more information, you can visit our Privacy Policy (https://es.gsk.com/media/771837/privacy-note.pdf). which provides more detailed and complete information on the processing we carry out on personal data.

We remind you of the possibility of exercising your rights of access, rectification, deletion, opposition, portability, limitation of processing, and not to be subject to automated decisions, which you may exercise by writing to "GSK", through the email address arco-general@gsk.com.

If you would like to learn more about Outcomes'10's privacy policy, please visit the https://www.outcomes10.com/politica-privacidad/

If you consider that the processing does not comply with current Data Protection regulations, you may file a complaint with the supervisory authority: Spanish Data Protection Agency (https://www.aepd.es).

By activating the check to read and accept our survey, you are agreeing to participate in this study, with all the conditions described in this document.

For technical support on the platform, please contact:

*This Site is not dedicated to the communication of adverse reactions or other safety information. If you wish to report any suspected adverse reaction or other safety information, you can do so through the corresponding Regional Pharmacovigilance Centre, through the yellow card system (*http://www.aemps.gob.es/vigilancia/medicamentosUsoHumano/docs/dir_serfv.pdf*), through the electronic form available on* https://www.notificaRAM.es *or through the laboratory that markets the product.*

DELPHI QUESTIONNAIRE

A. SOCIODEMOGRAPHIC VARIABLES

<table>
<tr><th colspan="3">SOCIODEMOGRAPHIC VARIABLES HEALTH PROFESSIONAL</th></tr>
<tr><td colspan="3">Age __________ years</td></tr>
<tr><td colspan="3">Years of experience managing rheumatology patients:
☐ Junior faculty (<15)
☐ Intermediate (15-30)
☐ Senior faculty (>30)</td></tr>
<tr><td colspan="3">Gender:
☐ Man
☐ Woman</td></tr>
<tr><td>AUTONOMOUS REGION health care facility where you practice:</td><td>☐Andalucía
☐Aragón
☐Asturias
☐Baleares
☐Canarias
☐Cantabria
☐Castilla-La Mancha
☐Castilla y León
☐Cataluña</td><td>☐Comunidad Valenciana
☐Extremadura
☐Galicia
☐La Rioja
☐Madrid
☐Murcia
☐Navarra
☐País Vasco
☐Ceuta y Melilla</td></tr>
<tr><td colspan="2">Please indicate your specialty:
☐ Rheumatology
☐ Nephrology
☐ Internal Medicine
☐ Dermatology</td><td>☐ Hospital Pharmacy
☐ Nursing
☐ Psychology</td></tr>
</table>

**Do you practice your specialty in a systemic lupus erythematosus monographic consultation?**<br>☐ Yes<br>☐ No
**Do you practice your specialty in a CSUR or XUECs reference center?**<br>☐ Yes<br>☐ No
**Time of experience in managing the patient with systemic lupus erythematosus** ______________________ years

Please indicate your degree of agreement with the following statements based on your experience in managing the patient with systemic lupus erythematosus:

B. USE OF PRO/PROMS IN CLINICAL PRACTICE

On April 19, 2022, a focus group was conducted with 9 patients and the caregiver of a 15-year-old patient (mean age 46.4 years). 78% women. A mean evolution time of 26.3 years (SD: 14.51). In this meeting, the aspects of greatest importance for patients to be included in the consultation were identified. In the symptom sphere, patients highlighted from most to least importance: disease activity (symptoms such as fatigue, morning stiffness, lupus haze, sleep disturbances), joint pain, inflammation, sleepiness, general pain, and fatigue. Meanwhile, in the sphere of impact of the disease, quality of life, emotional impact and functional capacity (variables with the highest score) stood out.

On June 9, 2022, these results were analysed by the scientific committee and a series of minimum domains were established to be considered for the most relevant PROM assessment in the follow-up of patients with SLE in clinical practice:

- Pain
- Fatigue
- Sleep disturbances: Difficulty sleeping or frequent awakenings
- Cognitive impairments: Functioning of one or more mental functions (e.g., lupus haze)
- Quality of life: An individual's physical, emotional, and social health
- Functional capacity: Limitation to perform day-to-day activities
- Emotional state: Includes anxiety and depression
- Work productivity: Ability to work or study
- Irreversible organ damage as a result of the disease
- Satisfaction with treatment: Includes acceptability and perceived benefit with the medication

To assess these domains, a guided review of the literature was carried out and those PROMs of free access and validated in Spanish were proposed and included in the following table and ordered from least to most complex:

PROMs (questionnaires)	Domains	Number of items	Rank	Time to Complete	Training	Interpretation
**DIMENSIONAL**						
**Visual Analogic Scale (VAS 0-100mm)**	Pain, fatigue, quality of life	Scales from 0-100mm	0-10	1 min	No	0 (not present) 100 (maximum possible)
**EuroQoL-5 Dimensions (EQ-5D)**	Quality of life	6	1-3<br>0-100	<2 minutes	No	1- No problems, 3- Extreme problems. EQ-5D VAS 0 worst health, 100 best health
**Lupus Impact Tracker (LIT*)**	Quality of life	10	0-100	<2 minutes	No	Higher scores indicate a greater negative impact of SLE
**Hospital Anxiety and Depression Scale (HADS)**	Emotional state	14	0–42	5 min	No	Higher scores indicate greater severity
**Functional Assessment of Chronic Illnes Therapy Fatigue (FACIT-Fatigue)**	Fatigue	13	0-52	3-4 minutes	No	High scores mean less fatigue
**Fatigue Severity Scale (FSS)**	Fatigue	9	9-63	n.e	No	Scores: 9 (no fatigue) 63 (maximum fatigue)
**Work Productivity and Activity Impairment: Lupus (WPAI: Lupus*)**	Labor Productivity	6	n.e	n.e	n.e	A higher score indicates a greater impact on health
**Oviedo Sleep Questionnaire**	Sleep disturbances	15	15-77	n.e	n.e	Higher score, higher severity of insomnia
**Pittsburg Quality of Sleep Inventory**	Sleep disturbances	19	0-21	5-10 minutes	No	Higher Scores Indicate Poorer Sleep Quality
**Treatment Satisfaction Questionnaire for Medication (TSQM)**	Satisfaction with treatment	14	0-100	n.e	n.e	The higher the score, the higher the patient satisfaction with medication
**Lupus Damage Index Questionnaire (LDIQ*)**	Irreversible damage (of 12 organs)	56	0-22	10 min	No	The higher the score, the greater the damage
**MULTIDIMENSIONAL**						
**LupusPRO**^	Functional capacity, pain, quality of life, emotional state, cognitive alterations	44	0-100	n.e	No	Higher Scores Mean Better Health
**Multi-Dimensional Health Assessment Questionnaire (MDHAQ)**	Functional capacity, pain, fatigue, emotional state	10	0-3	<5 minutes	No	Higher scores indicate poorer function and greater disability

*LES-specific PROMs. ^PROM to be considered when validated in Spain. N.E (unspecified)

BLOCK A. GENERAL QUESTIONS

1. Incorporating the patient's perspective through the **use of PROMs** in clinical practice **contributes to improving the management of patients** with SLE

	1	2	3	4	5	6	7
	Strongly disagree	Mostly disagree	Somewhat disagree	Neutral	Somewhat agree	Mostly agree	Strongly agree
	☐	☐	☐	☐	☐	☐	☐

2. In your opinion, consider whether it is necessary to implement the following measures to promote the use of PROMs in clinical practice:

a) Have a support professional in addition to the doctor to help the patient complete the PROMs

	1	2	3	4	5	6	7
	Strongly disagree	Mostly disagree	Somewhat disagree	Neutral	Somewhat agree	Mostly agree	Strongly agree
	☐	☐	☐	☐	☐	☐	☐

b) Have a digital tool that allows the patient to complete these questionnaires and can connect the results of the PROMs with the electronic medical record

	1	2	3	4	5	6	7
	Strongly disagree	Mostly disagree	Somewhat disagree	Neutral	Somewhat agree	Mostly agree	Strongly agree
	☐	☐	☐	☐	☐	☐	☐

BLOCK B. SPECIFIC QUESTIONS ABOUT PROMS

You will find a series of statements related to the use of each of the PROMs proposed in the table and their use in clinical practice.

We ask you to indicate your degree of agreement on each of these statements from three perspectives:

- Current situation: whether this PROM is currently used in your clinical practice or your environment
- Adequacy: whether you consider it appropriate to use this PROM to evaluate the domains it includes
- Feasibility: whether in your opinion it is feasible to use this PROM in clinical practice with the resources currently available.

3. **Visual Analog Scale (VAS 0-100mm)** can be used to assess pain, fatigue and the general condition of the patient with SLE. Below, we ask you to assess the use of VAS in each of these three aspects of the disease:

3a) PAIN:

	1	2	3	4	5	6	7
	Strongly disagree	Mostly disagree	Somewhat disagree	Neutral	Somewhat agree	Mostly agree	Strongly agree
**Current situation**	□	□	□	□	□	□	□
**Adequacy**	□	□	□	□	□	□	□
**Feasibility**	□	□	□	□	□	□	□

3b) FATIGUE:

	1	2	3	4	5	6	7
	Strongly disagree	Mostly disagree	Somewhat disagree	Neutral	Somewhat agree	Mostly agree	Strongly agree
**Current situation**	□	□	□	□	□	□	□
**Adequacy**	□	□	□	□	□	□	□
**Feasibility**	□	□	□	□	□	□	□

3c) GLOBAL ASSESSMENT OF THE DISEASE BY THE PATIENT:

	1	2	3	4	5	6	7
	Strongly disagree	Mostly disagree	Somewhat disagree	Neutral	Somewhat agree	Mostly agree	Strongly agree
**Current situation**	□	□	□	□	□	□	□
**Adequacy**	□	□	□	□	□	□	□
**Feasibility**	□	□	□	□	□	□	□

4. EuroQoL-5 Dimensions **Generic Quality of Life Questionnaire (EQ-5D)** assesses the **pain, quality of life, functionality and emotional state** of the patient with SLE.

	1	2	3	4	5	6	7
	Strongly disagree	Mostly disagree	Somewhat disagree	Neutral	Somewhat agree	Mostly agree	Strongly agree
**Current situation**	□	□	□	□	□	□	□
**Adequacy**	□	□	□	□	□	□	□
**Feasibility**	□	□	□	□	□	□	□

5. **Lupus Impact Tracker (LIT)** evaluates the **quality of life** of the patient with SLE.

	1	2	3	4	5	6	7
	Strongly disagree	Mostly disagree	Somewhat disagree	Neutral	Somewhat agree	Mostly agree	Strongly agree
**Current situation**	□	□	□	□	□	□	□
**Adequacy**	□	□	□	□	□	□	□
**Feasibility**	□	□	□	□	□	□	□

6. **Hospital Anxiety and Depression Scale (HADS), the** emotional state **of the patient with SLE** is assessed.

	1	2	3	4	5	6	7
	Strongly disagree	Mostly disagree	Somewhat disagree	Neutral	Somewhat agree	Mostly agree	Strongly agree
**Current situation**	□	□	□	□	□	□	□
**Adequacy**	□	□	□	□	□	□	□
**Feasibility**	□	□	□	□	□	□	□

7. **Functional Assessment of Chronic Illnes Therapy – Fatigue (FACIT-Fatigue),** evaluates the **fatigue** of patients with SLE.

	1	2	3	4	5	6	7
	Strongly disagree	Mostly disagree	Somewhat disagree	Neutral	Somewhat agree	Mostly agree	Strongly agree
**Current situation**	□	□	□	□	□	□	□
**Adequacy**	□	□	□	□	□	□	□
**Feasibility**	□	□	□	□	□	□	□

8. **Fatigue Severity Scale (FSS),** which assesses the **fatigue** of the patient with SLE.

	1	2	3	4	5	6	7
	Strongly disagree	Mostly disagree	Somewhat disagree	Neutral	Somewhat agree	Mostly agree	Strongly agree
**Current situation**	□	□	□	□	□	□	□
**Adequacy**	□	□	□	□	□	□	□
**Feasibility**	□	□	□	□	□	□	□

9. **Work Productivity and Activity Impairment: Lupus (WPAI: Lupus)** assesses the **work productivity** of patients with SLE.

	1	2	3	4	5	6	7
	Strongly disagree	Mostly disagree	Somewhat disagree	Neutral	Somewhat agree	Mostly agree	Strongly agree
**Current situation**	□	□	□	□	□	□	□
**Adequacy**	□	□	□	□	□	□	□
**Feasibility**	□	□	□	□	□	□	□

10. **Oviedo Sleep Questionnaire**, sleep disturbances **of patients with SLE** are evaluated.

	1	2	3	4	5	6	7
	Strongly disagree	Mostly disagree	Somewhat disagree	Neutral	Somewhat agree	Mostly agree	Strongly agree
**Current situation**	□	□	□	□	□	□	□
**Adequacy**	□	□	□	□	□	□	□
**Feasibility**	□	□	□	□	□	□	□

11. **Treatment Satisfaction Questionnaire for Medication (TSQM version 1.4)** assesses **satisfaction with the treatment of** patients with SLE.

	1	2	3	4	5	6	7
	Strongly disagree	Mostly disagree	Somewhat disagree	Neutral	Somewhat agree	Mostly agree	Strongly agree
**Current situation**	□	□	□	□	□	□	□

**Adequacy**	◻	◻	◻	◻	◻	◻	◻
**Feasibility**	◻	◻	◻	◻	◻	◻	◻

12. **The Lupus Damage Index Questionnaire (LDIQ)** assesses the **irreversible damage** of the patient with SLE.

	**1**	**2**	**3**	**4**	**5**	**6**	**7**
	Strongly disagree	Mostly disagree	Somewhat disagree	Neutral	Somewhat agree	Mostly agree	Strongly agree
**Current situation**	◻	◻	◻	◻	◻	◻	◻
**Adequacy**	◻	◻	◻	◻	◻	◻	◻
**Feasibility**	◻	◻	◻	◻	◻	◻	◻

13. **The Multi-Dimensional Health Assessment Questionnaire (MDHAQ) assesses the functional capacity, pain, fatigue, disease activity, and emotional state** of the patient with SLE.

	**1**	**2**	**3**	**4**	**5**	**6**	**7**
	Strongly disagree	Mostly disagree	Somewhat disagree	Neutral	Somewhat agree	Mostly agree	Strongly agree
**Current situation**	◻	◻	◻	◻	◻	◻	◻
**Adequacy**	◻	◻	◻	◻	◻	◻	◻
**Feasibility**	◻	◻	◻	◻	◻	◻	◻

14. Pittsburgh Quality of Sleep Inventory (PQSI) **questionnaire**, which evaluates sleep **disturbances** in patients with SLE.

	**1**	**2**	**3**	**4**	**5**	**6**	**7**
	Strongly disagree	Mostly disagree	Somewhat disagree	Neutral	Somewhat agree	Mostly agree	Strongly agree
**Current situation**	◻	◻	◻	◻	◻	◻	◻
**Adequacy**	◻	◻	◻	◻	◻	◻	◻
**Feasibility**	◻	◻	◻	◻	◻	◻	◻

15. **LupusPRO,** functional **capacity, pain, quality of life, emotional state, and cognitive alterations** of patients with SLE are evaluated. (Please note that it is translated into Spanish with population in Latin America, it is not validated in Spanish population)

	**1**	**2**	**3**	**4**	**5**	**6**	**7**
	Strongly disagree	Mostly disagree	Somewhat disagree	Neutral	Somewhat agree	Mostly agree	Strongly agree
**Current situation**	◻	◻	◻	◻	◻	◻	◻
**Adequacy**	◻	◻	◻	◻	◻	◻	◻
**Feasibility**	◻	◻	◻	◻	◻	◻	◻

C. MULTIDISCIPLINARY MANAGEMENT OF SLE PATIENTS

16. Multidisciplinary **consultations** make it possible to optimize the approach to patients living with SLE by facilitating and speeding up interaction/coordination between different specialties.

	**1**	**2**	**3**	**4**	**5**	**6**	**7**

	Strongly disagree	Mostly disagree	Somewhat disagree	Neutral	Somewhat agree	Mostly agree	Strongly agree
**Current situation**	□	□	□	□	□	□	□
**Adequacy**	□	□	□	□	□	□	□
**Feasibility**	□	□	□	□	□	□	□

17. The **unification of complementary tests** facilitates care and improves the quality of patient care (reducing duplication for the clinician, patient, etc.)

	1	2	3	4	5	6	7
	Strongly disagree	Mostly disagree	Somewhat disagree	Neutral	Somewhat agree	Mostly agree	Strongly agree
**Current situation**	□	□	□	□	□	□	□
**Adequacy**	□	□	□	□	□	□	□
**Feasibility**	□	□	□	□	□	□	□

18. The **incorporation of hospital pharmacists into the care team that manages the patient with SLE would improve the management of medication in the patient with SLE (**e.g., avoiding possible medication interactions, etc.)

	1	2	3	4	5	6	7
	Strongly disagree	Mostly disagree	Somewhat disagree	Neutral	Somewhat agree	Mostly agree	Strongly agree
**Current situation**	□	□	□	□	□	□	□
**Adequacy**	□	□	□	□	□	□	□
**Feasibility**	□	□	□	□	□	□	□

19. **Telepharmacy programs** optimize patient care in the outpatient setting, reducing the need for the patient to travel to the hospital pharmacy for medication dispensing and follow-up.

	1	2	3	4	5	6	7
	Strongly disagree	Mostly disagree	Somewhat disagree	Neutral	Somewhat agree	Mostly agree	Strongly agree
**Current situation**	□	□	□	□	□	□	□
**Adequacy**	□	□	□	□	□	□	□
**Feasibility**	□	□	□	□	□	□	□

D. PATIENT EMPOWERMENT

20. Patient associations could play an important role in informing and training patients **on the use of PRO/PROM and their incorporation into clinical practice**.

	1	2	3	4	5	6	7
	Strongly disagree	Mostly disagree	Somewhat disagree	Neutral	Somewhat agree	Mostly agree	Strongly agree
**Current situation**	□	□	□	□	□	□	□
**Adequacy**	□	□	□	□	□	□	□
**Feasibility**	□	□	□	□	□	□	□

21. In addition to the physician responsible for patient follow-up, **nursing** has a key role in training **and educating the patient for the use of PRO/PROMs**.

	**1**	**2**	**3**	**4**	**5**	**6**	**7**
	Strongly disagree	Mostly disagree	Somewhat disagree	Neutral	Somewhat agree	Mostly agree	Strongly agree
**Current situation**	□	□	□	□	□	□	□
**Adequacy**	□	□	□	□	□	□	□
**Feasibility**	□	□	□	□	□	□	□

22. PROMs can facilitate patient participation in decision-making and thus **improve treatment adherence.**

	**1**	**2**	**3**	**4**	**5**	**6**	**7**
	Strongly disagree	Mostly disagree	Somewhat disagree	Neutral	Somewhat agree	Mostly agree	Strongly agree
**Current situation**	□	□	□	□	□	□	□
**Adequacy**	□	□	□	□	□	□	□
**Feasibility**	□	□	□	□	□	□	□

Below you can add any comments or suggestions not included in this survey, which you consider important

Comment:
