# Supplementary material for: Patient-reported outcome measures for systemic lupus erythematosus: an expert Delphi consensus to guide implementation in routine care
Source: BMC Rheumatol. 2024 Jul 16;8:31. doi: 10.1186/s41927-024-00401-x (PMC11251319; doi:10.1186/s41927-024-00401-x)
Supplement: Supplementary file 3 — Supplementary Material 3. [file 41927_2024_401_MOESM3_ESM.pdf]

Supplementary File 3: Delphi patient questionnaire focused on the current management of the patient living with systemic lupus erythematosus and the current challenges in clinical practice.

INFORMATION SHEET

You have been invited to participate in the project **"Current management of patients living with systemic lupus erythematosus. Current Challenges in Clinical Practice".**

This project is advised by the scientific committee formed by:

Name	Service / Hospital
**Isabel Castrejón (Project Coordinator)**	Rheumatology Department. Hospital Gregorio Marañón (Madrid)
**Maria Galindo**	Rheumatology Department. Hospital 12 de octubre (Madrid)
**Alejandro Muñoz**	Rheumatology Department. Hospital Virgen del Rocío (Sevilla)
**María Jose Cuadrado**	Rheumatology Department. Hospital Clínica Universitaria de Navarra (Madrid)
**Tarek Salman**	Rheumatology Department. Hospital del Mar (Barcelona)
**Joaquín Borrás**	Hospital Pharmacy Service. Hospital de Sagunto (Valencia)
**Laura Cano**	Nursing Rheumatology. Hospital Regional de Málaga (Málaga)

WHAT IS THE GOAL OF THE PROJECT?

The objective of the project is to reach a consensus and propose strategies to promote the implementation of patient-reported variables (PRO) such as pain, fatigue, sleep disturbances, quality of life, etc., and their measurement instruments (PROM) in the follow-up of patients with systemic lupus erythematosus in consultation.

To this end, different health professionals (rheumatology, nephrology, internal medicine, dermatology, hospital pharmacy, nursing and psychology) involved in the care of patients with lupus will participate in the project.

WHAT DOES YOUR PARTICIPATION INVOLVE?

Your participation will consist of responding to **two rounds of Delphi** consultation through a **questionnaire in electronic format**, the completion of which will take a **maximum of 10 minutes**.

The Delphi **consensus first round questionnaire** presents a series of questions written as statements, where you must indicate **your degree of agreement, based on your experience** in day-to-day experience with systemic lupus erythematosus, usinga 7-point Likert scale where 1= "strongly disagree" and 7= "strongly agree".

In the **second round of Delphi consultation**, only those statements that do not reach consensus in the first round or aspects proposed by the participants and not considered in the first round will be presented. The objective of this second round is to reach consensus on the answers and, therefore, the second questionnaire will be personalized for each participant, including the overall and individual score obtained in the first round.

At the end of the questionnaire you will have a free text section where you can make the comments you consider appropriate or clarify the aspects you consider relevant. In addition, you will be able to indicate aspects that are important to you and not included in the Delphi questionnaire, which may be incorporated into the second round if the scientific committee deems it appropriate.

CONFIDENTIALITY

Data protection

In compliance with data protection regulations, in particular Regulation (EU) 2016/679, of the European Parliament and of the Council, of 27 April 2016, we inform you that the personal data you provide us through this survey will be incorporated into the files and systems under the responsibility of "Glaxosmithkline SA (GSK) CIF-A28228526" will be used for communications related to the study "Current Management of the Patient Living with Systemic Lupus Erythematosus. Current Challenges in Clinical Practice".

Your data has been transferred to OUTCOMES'10 in order to carry out the study, as well as the necessary communications during the process of carrying it out, being deleted after the publication of the manuscript, within the legal deadlines established for this purpose according to current legislation. Outcomes'10 guarantees the adoption of the necessary technical and organisational measures to ensure the confidential treatment of personal data. There are no plans to transfer data. We inform you that international transfers are not made outside the European Economic Area. And that this processing does not imply the existence of automated decision-making or profiling.

Categories of Personal Data: General Population

The basis for legitimising the processing is the consent of the data subject (Article 6(1)(a) GDPR).

For more information, you can visit our Privacy Policy (https://es.gsk.com/media/771837/privacy-note.pdf). which provides more detailed and complete information on the processing we carry out on personal data.

We remind you of the possibility of exercising your rights of access, rectification, deletion, opposition, portability, limitation of processing, and not to be subject to automated decisions, which you may exercise by writing to "GSK", through the email address arco-general@gsk.com.

If you would like to learn more about Outcomes'10's privacy policy, please visit the https://www.outcomes10.com/politica-privacidad/

If you consider that the processing does not comply with current Data Protection regulations, you may file a complaint with the supervisory authority: Spanish Data Protection Agency (https://www.aepd.es).

By activating the check to read and accept our survey, you are agreeing to participate in this study, with all the conditions described in this document.

For technical support on the platform, please contact:

*This Site is not dedicated to the communication of adverse reactions or other safety information. If you wish to report any suspected adverse reaction or other safety information, you can do so through the corresponding Regional Pharmacovigilance Centre, through the yellow card system (*http://www.aemps.gob.es/vigilancia/medicamentosUsoHumano/docs/dir_serfv.pdf*), through the electronic form available on* https://www.notificaRAM.es *or through the laboratory that markets the product.*

DELPHI QUESTIONNAIRE

A. SOCIODEMOGRAPHIC VARIABLES

PATIENT SOCIODEMOGRAPHIC VARIABLES		
**Age** __________ years		
**Gender:**<br>☐ Man<br>☐ Woman		
**AUTONOMOUS COMMUNITY to which the health centre where you work belongs:**<br>**(drop-down)**	☐Andalucía<br>☐Aragón<br>☐Asturias<br>☐Baleares<br>☐Canarias<br>☐Cantabria<br>☐Castilla-La Mancha<br>☐Castilla y León<br>☐Cataluña	☐Comunidad Valenciana<br>☐Extremadura<br>☐Galicia<br>☐La Rioja<br>☐Madrid<br>☐Murcia<br>☐Navarra<br>☐País Vasco<br>☐Ceuta y Melilla
**Time since diagnosis of Systemic Lupus Erythematosus** ______________________ Years		

Please indicate your degree of agreement with the following statements based on your experience as a patient with systemic lupus erythematosus. There are no true or false answers, we simply want to know your opinion as a patient on different aspects of the disease (you can find an explanatory comment in each statement):

B. USE OF PRO/PROMS IN CONSULTATION

You will find a number of statements related to the use of these PROMs in consultation. We ask you to indicate your degree of agreement on each of these statements

1. Incorporating your perspective through questionnaires specifically developed to assess the perspective of patients with SLE (hereinafter **PROMs)** in consultation **contributes to a better management of your disease.**

	1	2	3	4	5	6	7
	Strongly disagree	Mostly disagree	Somewhat disagree	Neutral	Somewhat agree	Mostly agree	Strongly agree
	☐	☐	☐	☐	☐	☐	☐

2. In your opinion, please consider whether it is necessary to implement the following measures to promote the use of PROMs in consultation:

a) Have a support professional in addition to the doctor to help the patient complete the questionnaires.

	1	2	3	4	5	6	7
	Strongly disagree	Mostly disagree	Somewhat disagree	Neutral	Somewhat agree	Mostly agree	Strongly agree
	☐	☐	☐	☐	☐	☐	☐

b) Have a digital tool that allows the patient to complete these questionnaires and can connect the results of the PROMs with the electronic medical record.

	1	2	3	4	5	6	7
	Strongly disagree	Mostly disagree	Somewhat disagree	Neutral	Somewhat agree	Mostly agree	Strongly agree
	☐	☐	☐	☐	☐	☐	☐

C. MULTIDISCIPLINARY MANAGEMENT OF SLE PATIENTS

For each statement, you will need to consider three perspectives:

- Current situation (if you have experienced it when you have gone to consultations)
- Adequacy (if deemed appropriate/relevant for follow-up in consultations)
- Feasibility (if you think this could be made available in consultation with current resources)

3. Multidisciplinary **consultations** improve the care of patients living with SLE by facilitating interaction between different specialists.

	1	2	3	4	5	6	7

	Strongly disagree	Mostly disagree	Somewhat disagree	Neutral	Somewhat agree	Mostly agree	Strongly agree
**Current situation**	◻	◻	◻	◻	◻	◻	◻
**Adequacy**	◻	◻	◻	◻	◻	◻	◻
**Feasibility**	◻	◻	◻	◻	◻	◻	◻

4. The **unification of complementary tests** facilitates care and improves the quality of patient care (reducing duplication for the clinician, patient, etc.)

	**1**	**2**	**3**	**4**	**5**	**6**	**7**
	Strongly disagree	Mostly disagree	Somewhat disagree	Neutral	Somewhat agree	Mostly agree	Strongly agree
**Current situation**	◻	◻	◻	◻	◻	◻	◻
**Adequacy**	◻	◻	◻	◻	◻	◻	◻
**Feasibility**	◻	◻	◻	◻	◻	◻	◻

5. **Telematic dispensing of medication** (telepharmacy) programs improve patient care in the outpatient clinic, reducing the need to travel to the hospital pharmacy to pick up medication.

	**1**	**2**	**3**	**4**	**5**	**6**	**7**
	Strongly disagree	Mostly disagree	Somewhat disagree	Neutral	Somewhat agree	Mostly agree	Strongly agree
**Current situation**	◻	◻	◻	◻	◻	◻	◻
**Adequacy**	◻	◻	◻	◻	◻	◻	◻
**Feasibility**	◻	◻	◻	◻	◻	◻	◻

D. PATIENT EMPOWERMENT

6. Patient associations could play an important role in informing and training **patients in the use of the aforementioned questionnaires (PRO/PROM) and their incorporation into** consultations.

	**1**	**2**	**3**	**4**	**5**	**6**	**7**
	Strongly disagree	Mostly disagree	Somewhat disagree	Neutral	Somewhat agree	Mostly agree	Strongly agree
**Current situation**	◻	◻	◻	◻	◻	◻	◻
**Adequacy**	◻	◻	◻	◻	◻	◻	◻
**Feasibility**	◻	◻	◻	◻	◻	◻	◻

You can then add other forms of information or training that you consider more appropriate

Comment:

7. PROMs can facilitate patient participation in decision-making and thus **improve treatment adherence.**

	**1**	**2**	**3**	**4**	**5**	**6**	**7**

	Strongly disagree	Mostly disagree	Somewhat disagree	Neutral	Somewhat agree	Mostly agree	Strongly agree
**Current situation**	☐	☐	☐	☐	☐	☐	☐
**Adequacy**	☐	☐	☐	☐	☐	☐	☐
**Feasibility**	☐	☐	☐	☐	☐	☐	☐

Below you can add any comments or suggestions not included in this survey, which you consider important

Comment:
